# Predicting Adherence to Walking from Anxiety, Depression, Disease Impact, Catastrophizing, and Cognitive Fusion in Patients with Fibromyalgia: Does Pain Severity Matter?

**DOI:** 10.3390/ijerph192416453

**Published:** 2022-12-08

**Authors:** Patricia Catala, Carmen Écija, Angel Serrano del Moral, Estibalitz Perez Viejo, Cecilia Peñacoba

**Affiliations:** 1 Department of Psychology, Rey Juan Carlos University, Avda. de Atenas s/n, 28922 Madrid, Spain; 2 General Surgery and Digestive Surgery Service, Hospital Universitario de Fuenlabrada, 28944 Madrid, Spain

**Keywords:** adherence to walking, pain severity, fibromyalgia, psychological processes, moderation

## Abstract

Aim: This study analyzed whether the contribution of several factors associated with walking adherence in fibromyalgia (FM) patients varies across pain severity levels. Methods: Participants were 228 women with FM (mean age 57 years; SD = 8.49). Results: Bivariate analyses replicated the expected association between predictors (FM impact, anxiety, depression, catastrophizing, and cognitive fusion) and poorer adherence to walking. Multivariate analyses showed a negative contribution of FM impact, catastrophizing, and depression on walking adherence after controlling for pain levels (all *p* < *0*.01). A moderation effect of pain severity in the relationship between predictors and adherence to walking was only found for cognitive fusion (*B* = −0.01, *t* = −2.02, *p* = 0.040). Specifically, cognitive fusion only contributed to poor walking adherence at moderate and severe pain levels, but not when pain was mild. The contribution of the remaining predictors was not moderated by pain levels, which means that they contributed to walking adherence irrespective of the pain severity of the patient. Pain severity did not contribute to walking adherence when controlling for the predictors. Conclusion: Clinical implications are discussed from the perspective of personalized interventions and preferable target interventions when attempting to increase adherence to walking in this population.

## 1. Introduction

Fibromyalgia (FM) is considered one of the most frequent, expensive, and, also importantly, disabling chronic pain problems globally [1]. Indeed, this disability partly has a crucial role in this population as patients with FM often reduce their participation in physical activities dramatically in an attempt to control their symptomatology, which in turn exacerbates the severity of the condition in a type of vicious cycle [2,3].

Physical therapy is one of the interventions of choice for this population because it can mitigate some of the symptoms associated with the disease (e.g., muscle inflexibility, muscle tension, and imbalance) and can help gain overall strength and maintain range of motion, thus reducing disability [4,5]. In particular, walking is a frequently recommended aerobic physical exercise [6] because of its low musculoskeletal impact [7] and known benefits for pain management, physical functionality, and improved mood [7]. Discouragingly, research shows that FM patients often do not adhere to the recommendations on physical activity, including walking [8,9], as they often attribute exercising to increased pain severity when performing the activity [4].

Compliance with physical exercise has been a matter of concern for decades, and addressing compliance problems in physical exercise treatment programs should be a mandatory requirement according to the literature [10]. Traditionally, long-term, unsupervised compliance with physical exercise, which should be the focus of interest in chronic conditions such as FM, has been argued to be influenced by a combination of several factors in the individual, such as perceived illness, coping skills, and appraisals [11,12]. Therefore, in the present investigation, we will evaluate whether adherence to walking in a sample of FM patients is indeed associated with a set of important factors in the individual. Specifically, FM impact on functioning, depressive symptoms, anxiety, catastrophizing (cognitive bias that leads us to imagine the worst possible scenarios, which leads us to feed a series of irrational beliefs that end up affecting our attitudes, behaviors, and decisions), and cognitive fusion (tendency to believe the literal content of thought and feeling, the excessive or improper regulation of behavior by verbal processes, rather than by environmental contingencies) will be taken into account. Different to past research, however, we will explore whether the contribution of these variables on adherence to walking is indeed influenced by pain severity levels (i.e., moderation) as indicated by patients [4]. This is important for personalized interventions (i.e., whether the same recommendations and treatment plans can be proposed to enhance adherence to walking irrespective of pain severity status).

This moderation effect of pain severity was already evidenced in past research showing that the relationship between pain catastrophizing and physical health status and pain interference is reduced as pain levels increase, which was attributed to the relatively inescapable nature of pain when this is very severe [13]. The extent to which this is also true for adherence to walking remains unexplored and is the main goal of the present investigation. Based on past research, we expect that FM impact, depressive symptoms, anxiety, catastrophizing, and cognitive fusion will be associated with poor adherence to walking, and we anticipate that this relationship will be stronger at lower levels of pain (i.e., moderation).

## 2. Materials and Methods

### 2.1. Participants

Participants were 228 women diagnosed with FM according to the criteria of the American College of Rheumatology [14] recruited from different associations in Spain. Once the participants gave their informed consent to participate in the study, they were given a questionnaire booklet that took approximately 30 min to complete. The study followed the ethical principles for research with human participants and was approved by the University Ethics Committee (Universidad Rey Juan Carlos, Reference number: PI17/00858).

### 2.2. Measures

Pain Severity: The Brief Pain Inventory (BPI) [15] was used to assess average pain intensity, as recommended in clinical guidelines [16]. BPI is made up of 9 items that are arranged into two components: pain intensity and pain interference (with general activities, mood, ability to walk, normal work, relationships with others, sleep, and enjoyment of life). Specifically, for the present study, the 4 items that refer to pain intensity were used: during the past 24 h (2 items: worst and least), average pain (1 item) and current pain (1 item). Item labels range from 0 = “no pain” to 10 = “worst possible pain” and are calculated using the mean of all four items, in such a way that the range of scores of this variable oscillates between 0 and 10. A high score represents high pain intensity. Cronbach’s alpha in this study was 0.89.

Impact of fibromyalgia on functioning: The total score of the Spanish adaptation of the Fibromyalgia Impact Questionnaire–Revised was used to measure the perceived impact of FM on functioning [17]. This questionnaire is made up of 21 items that are answered on a numerical rating scale of 11 points ranging from 0 to 10, with 10 being “worst”. Items have different verbal anchors. The FIQR is divided into three domains: function, general impact, and symptoms. The total FIQR is the sum of the three modified domain scores. The summed score for function (range 0–90) is divided by 3; the summed score for overall impact (range 0–20) does not change; and the summed score for symptoms (range 0–100) is divided by 2. Higher scores represent a greater perceived impact of FM on performance, with 100 being the maximum score. The Cronbach’s alpha of the scale in the present study was 0.88.

Anxiety and depression: The Spanish adaptation of the Hospital Anxiety and Depression Scale was used in the present study [18]. This is a 14-item scale, in which 7 assess anxiety and 7 evaluate depression. Items are responded to on a 4-point Likert scale that varies from 0 = “no, not at all” to 3 = “yes, definitely”. A higher score represents higher levels of anxiety and depression, with 21 being the maximum score. Internal consistency estimates in the present study were good in both domains (0.82 in anxiety and 0.85 in depression).

Pain catastrophizing: The Spanish adaptation of the Pain Catastrophizing Scale was used [19]. The scale is made up of 13 items scored on a 5-point Likert scale from 0 (never) to 4 (always) and can be conceptualized as a combination of three different catastrophizing components (i.e., magnification, helplessness, and rumination) or a total catastrophizing score. The global score was preferred in the present study to reduce the number of statistical analyses, which minimizes the risk of false positive errors. The total score is obtained with the sum of the answers, being able to obtain a maximum score of 52. Higher scores in the scale represent a higher tendency to catastrophize. The total score had an alpha value of 0.88 in the present study.

Cognitive fusion: The Spanish adaptation of the Cognitive Fusion Questionnaire was used [20]. This questionnaire contains 7 items rated on a 7-point Likert scale ranging from 1 = “never” to 7 = “always”. The cognitive fusion score is obtained by summing up the score of the 7 items and ranges from 7 to 49. High scores indicate high cognitive fusion. In the current study, the Cronbach’s alpha was 0.86.

Walking behavior: An ad-hoc dichotomous (0 = ”no”; 1 = ”yes”) question was used to assess whether participants walked in order to exercise. Specifically, patients were asked about one of the recommended walking patterns for fibromyalgia: “to walk between 2 and 4 days a week, a minimum of 30 min per day, in bouts of 15–20 min, with a small rest between bouts over a minimum of six consecutive weeks” [7].

Sociodemographic and clinical data: An ad-hoc questionnaire was used to assess age, marital status, educational level, and employment status. Regarding clinical data, FM duration was also recorded.

### 2.3. Data Analysis

The SPSS 22 statistical package (IBM, Armonk, NY, USA) was used to perform the analyses [21]. After a descriptive analysis of sample characteristics, the bivariate associations between study variables (pain severity, walking, impact of FM, depression, anxiety, pain catastrophizing, and cognitive fusion,) were investigated. Pearson correlations were performed for continuous variables and student’s t-test for dichotomous variables (i.e., walking). Next, a series of multivariate regressions were computed with the PROCESS macro [22]. In each regression, a combination of one independent variable (i.e., disease impact, depression, anxiety, pain catastrophizing, or cognitive fusion), the moderator (i.e., pain severity), and their interaction were entered to predict the study outcome (i.e., walking). Then, when a significant moderation was found, the conditional effects were calculated (that is, the effects of an independent variable on a result for the different values of a moderator). For this, the pick-a-point approach was used, which establishes three levels of the moderator variable (low, medium, and high) that correspond to the 16th, 50^th^, and 84th percentiles of pain levels. These values are recommended for tables. In these post hoc analyses, non-centered variables were used to facilitate the interpretation of the results. The effect size was estimated using the coefficient of determination (R2) [23], having established the following quantification parameters: 0.02, 0.13, and 0.26, for small, medium, and large effect sizes, respectively [24]. In addition, the Johnson–Neyman technique was also used, which allows us to establish a greater range of the moderator variable in which to observe the effect of the variable X on Y. For each value of the moderator variable, a coefficient is calculated that quantifies the effect of X on Y. Specifically, it is observed at what level of the moderator variable this significant effect begins to be noticed.

## 3. Results

### 3.1. Sample Characteristics

The mean age of the participants was 56.91 years (SD = 8.94). In relation to the level of education, 24% of women reported having completed primary education, 61% secondary education, and 15% higher education. Fifty-three percent of the women were married or in a stable relationship; 11% were single; and 36% were divorced or widowed. In general, the participants were housewives (76%). The time range of diagnosis of FM in these women was from 1 to 46 years, and the average was 12.14 years (SD = 8.45).

### 3.2. Descriptive and Correlation Analysis

Table 1 shows the means, standard deviations, and Pearson correlations between study variables. Pain severity was positively associated with FM impact, anxiety, depression, pain catastrophizing, and cognitive fusion (all *p* < *0*.01). In addition, significant differences were observed between walking versus non-walking patients in the impact of FM (*t* = 3.53, *p* = 0.001), anxiety (*t* = 1.99, *p* = 0.040), depression (*t* = 3.57, *p* = 0.001), pain catastrophizing (t = 2.82, *p* = 0.005), and cognitive fusion (*t* = 2.73, *p* = 0.007). The patients who did not walk obtained higher scores in all the variables compared to those who walked. The effect size was small for pain intensity (Cohen’s d = 0.26), anxiety (Cohen’s d = 0.27), catastrophizing (Cohen’s d = 0.37), cognitive fusion (Cohen’s d = 0.37), and depression (Cohen’s d = 0.45), and medium for the impact of FM (Cohen’s d = 0.50).

### 3.3. Multivariate Linear Regression and Moderation Analyses

Table 2 shows the results of the regression analyses, including the moderations. The analyses revealed a significant contribution of FM impact (*B* = −0.09, *t* = −2.92, *p* = 0.003, 95% CI = 0.12, 0.71), depression (*B* = 0.35, *t* = −3.07, *p* = 0.002, 95% CI = 0.07, 0.62), pain catastrophizing (*B* = −0.03, *t* = −3.28, *p* = 0.001, 95% CI = −0.05, −0.01), and cognitive fusion (*B* = −0.04, *t* = −2.60, *p* = 0.009, 95% CI = −0.07, −0.01) in the prediction of walking. No direct effects of pain on adherence to walking were observed in any of the five regressions considered, that is, when pain intensity is included as a predictor together with fibromyalgia impact, anxiety, depression, pain catastrophizing, or cognitive fusion independently. Regarding the moderation analyses, the results revealed that the severity of the pain moderated the relationship between cognitive fusion and walking behavior (*B* = −0.01, *t* = −2.02, *p* = 0.040, 95% CI = −0.02, −0.01). The relationship between FM impact, depression, anxiety, pain catastrophizing, and walking was not moderated by the severity of pain (all *p* > *0*.05). That is, pain levels do not interfere in the relationships established between the study variables and walking behavior, except for cognitive fusion. In this case, cognitive fusion is related to walking depending on the levels of pain presented.

Conditional analyses were planned to explore significant moderations on cognitive fusion in depth. Results are presented in Table 3 and show that the contribution of cognitive fusion on walking behavior was only significant at moderate and severe levels of pain. The Johnson–Neyman technique allows us to add that from a value of 6.125 (*p* = 0.5) the effect of the variable (cognitive fusion) on walking begins to be significant. That is, from a pain level of 6.125, a negative and statistically significant effect of cognitive fusion on walking behavior is observed. In this sample, 61.16% of women are above this level of pain. Therefore, when the pain score is greater than or equal to 6.125, cognitive fusion interferes with walking behavior.

## 4. Discussion

The main objective of the present work was to identify a set of factors associated with walking adherence in a sample of patients with FM and to explore the moderating role of pain severity in this relationship. Past research emphasized the role of pain severity and pain-related emotional (i.e., anxiety, depression) and cognitive (i.e., pain catastrophizing, cognitive fusion) factors as predictors of physical functioning [25,26]. Research also suggested that the contribution of psychological factors on physical outcomes might differ as a function of pain levels [13]. Contrary to our expectations, the present study indicates that this is not the case for the functional, emotional, and cognitive processes contemplated in this study related to walking, with the exception of cognitive fusion. This might suggest that, for the most part, there is no need to adjust psychological treatments addressed to increase adherence to walking as a function of pain severity levels.

From our clinical experience and based on past research [4], patients often indicate that the risk of increasing pain levels largely explains their decision to adhere to exercising in general and walking in particular. However, the present study evidenced that pain severity levels are not associated with adherence to walking when controlling for the contribution of perceived functional status, emotional status, or cognitive processes (moderation analysis). Therefore and contrary to the frequent reports by patients, a focus on reducing or controlling pain severity levels only might not be sufficient to ensure adherence to walking in this population, and multidisciplinary and interdisciplinary approaches would be preferable [27].

In this sense, perceived FM impact, emotional status (i.e., severity of depressive symptomatology), and cognitive processes (i.e., catastrophizing and cognitive fusion) did predict poor adherence to walking, even after controlling for pain severity levels. This is consistent with existing research showing that the perceived illness status and psychological factors may play an important role in the physical performance of patients with FM [28,29,30] and, particularly, in their decision to exercise [11,12]. The results also provide further support for the fear-avoidance of the pain model of pain [31] and the psychological flexibility model of pain [32].

Specifically, according to the fear avoidance model, catastrophizing would lead to fear pain, thus boosting emotional distress and avoidance of activities that some might believe to increase pain, such as walking [31]. On the other hand, the psychological flexibility model emphasizes the role of cognitive fusion as a maladaptive form of relating with thoughts, memories, and feelings and argues that being fused with thoughts might lead to inactivity and distress because thoughts (e.g., “Walking will definitely increase my pain”) are considered to be truths that necessarily have to guide one’s behavior [32].

Our results are consistent with the aforementioned idea that certain cognitive processes might be associated with functional outcomes, particularly with adherence to walking. A novel finding was that the contribution of catastrophizing on walking occurred across pain severity levels which, contrary to past research [13], suggests that this cognitive process is likely to be maladaptive for the functional outcome of study (walking) irrespective of pain severity levels. An interesting finding, however, was that cognitive fusion only contributed to adherence to walking at moderate and severe levels of pain. This is consistent with the idea that pain might be an important contextual variable in the relationship between certain psychological factors and outcomes in this population [33]. Specifically, it suggests that, when pain is likely to be more attention-demanding (≥7 in an 11-point rating scale), not merging with the thoughts associated with pain is likely to be of upmost importance. When pain is less attention-capturing (≤5), the use of defusion techniques might be less relevant to determine adherence to walking. This opens interesting avenues for research and clinical work and provides new insights into the psychological flexibility model of pain.

In clinical practice, cognitive behavioral therapy has been the reference treatment to try to increase adherence to physical activity [10,34]. However, its efficacy was only demonstrated in the short-term [35] and showed a small effect in the medium-term [34]. In this sense, the reviews and meta-analyses carried out on adherence to treatment indicate the lack of theoretical knowledge by primary health professionals as the main reason for low adherence to exercising [10,36]. Bearing this in mind and taking into account the results found in the present study, it is first necessary to carry out an evaluation of the personal characteristics of the patients (e.g., tendency to catastrophize and get fused with thoughts, depressive symptomatology, and perceived functional status). Subsequently, and based on the results of this evaluation, professionals might want to provide motivational guidelines to patients with more at-risk profiles (high catastrophizing, fusion, depression, and low perceived functional status) either based on the fear of pain avoidance model or the psychological flexibility model. Based on the relationship between two of their main treatment target mechanisms (i.e., pain catastrophizing and cognitive fusion) involved in previous models (fear of pain avoidance and psychological flexibility), both might be relevant objectives when patients present low adherence to walking [10,34,37], but focus on pain catastrophizing would be preferred when patients present mild pain levels only.

There are several limitations to our study. First, this study presents a cross-sectional design. Therefore, cause-effect relationships cannot be established. Second, the results are obtained from self-report questionnaires; therefore, the results are subject to response bias. Third, since the results are based on women with FM (the most frequent gender in this condition), more research is needed on men and on other chronic pain populations to check whether the findings are generalizable.

## 5. Conclusions

The findings found here might have some important practical implications. As previously mentioned, pain severity has been thought to be an important factor predicting low adherence to walking. However, in long-term adherence to walking behaviors, pain seems to acquire less importance compared to other factors, such as perceived functionality, emotional state (depression), and cognitive profile (catastrophization and cognitive fusion). It seems also important, only for cognitive fusion, to take into account the patient’s pain level in order to address practice (the implementation of cognitive defusion might be preferred as pain increases). In all other cases, addressing the target variables might be equally relevant irrespective of pain levels. This is important for personalizing and increasing the efficacy of interventions.

## Figures and Tables

**Table 1:** Means, standard deviations, and Pearson correlations between study variables.

	Mean (SD)	2	3	4	5	6
1. Pain severity	6.88 (2.01)	0.52 ***	0.21 **	0.15 **	0.35 ***	0.20 **
2. FM impact	72.35 (17.00)		0.42 ***	0.51 ***	0.54 ***	0.45 ***
3. Anxiety	12.21 (3.86)			0.53 ***	0.44 ***	0.63 ***
4. Depression	9.22 (4.27)				0.44 ***	0.50 ***
5. Catastrophizing	31.80 (11.68)					0.45 ***
6. Cognitive fusion	33.30 (9.61)					
Walking	Yes = 58%					

*** *p* < 0.001, ** *p* < 0.01. FM, fibromyalgia.

**Table 2:** Prediction of adherence to walking from pain severity, study predictors (fibromyalgia impact, anxiety, depression, catastrophizing, and cognitive fusion), and their interaction (moderation analysis).

	−2LL	*p*	R^2^ CoxSneell	R^2^ Nagelkrk	Beta	*t*	*p*	95% CI
DV = Walking								
Model 1	290.32	0.001 ***	0.06	0.08				
Fibromyalgia impact					−0.09	−2.92	0.003 **	0.12, 0.71
Pain					−0.05	−0.66	0.505	−0.15, 0.03
Interaction					−0.01	−1.33	0.183	−0.03, 0.01
Model 2	297.71	0.044 *	0.03	0.05				
Anxiety					−0.05	−1.48	0.138	−0.12, 0.01
Pain					−0.12	−1.67	0.094	−0.26, 0.02
Interaction					−0.02	−1.31	0.188	−0.05, 0.01
Model 3	292.24	0.003 **	0.05	0.07				
Depression					0.35	−3.07	0.002 **	0.07, 0.62
Pain					−0.10	−1.45	0.145	−0.17, −0.03
Interaction					0.01	0.146	0.883	−0.02, 0.03
Model 4	296.73	0.028 *	0.04	0.05				
Pain catastrophizing					−0.03	−3.28	0.001 ***	−0.05, −0.01
Pain					0.01	0.03	0.976	−0.15, 0.16
Interaction					−0.01	−1.62	0.104	−0.01, 0.01
Model 5	290.36	0.002 **	0.06	0.08				
Cognitive fusion					−0.04	−2.60	0.009 **	−0.07, −0.01
Pain					−0.11	−1.54	0.121	−0.25, −0.03
Interaction					−0.01	−2.02	0.040 *	−0.02, −0.01

*** *p* < 0.001, ** *p* < 0.01, * *p* < 0.05.

**Table 3:** Conditional effects of cognitive fusion on walking at values of pain severity.

Pain Severity	Beta (Cognitive Fusion)	*t*	*p*	95% CI
5	−0.015	−0.81	0.414	−0.05, 0.02
7	−0.04	−2.68	0.007 **	−0.07, −0.01
9	−0.07	−3.03	0.002 **	−0.11, −0.02

** *p* < 0.01.

## Data Availability

The data presented in this study are available on request from the corresponding author. The data are not publicly available due to privacy restrictions.

## References

[1] Lacasse A., Bourgault P., Choinière M. (2016). Fibromyalgia-related costs and loss of productivity: A substantial societal burden. BMC Musculoskelet. Disord..

[2] Häuser W., Ablin J., Fitzcharles M.-A., Littlejohn G., Luciano J.V., Usui C., Walitt B. (2015). Fibromyalgia. Nat. Rev. Dis. Prim..

[3] Wolfe F., Clauw D.J., Fitzcharles M.-A., Goldenberg D.L., Häuser W., Katz R.L., Mease P.J., Russell A.S., Russell I.J., Walitt B. (2016). 2016 Revisions to the 2010/2011 fibromyalgia diagnostic criteria. Semin. Arthritis Rheum..

[4] Busch A.J., Webber S.C., Brachaniec M., Bidonde J., Bello-Haas V.D., Danyliw A.D., Overend T.J., Richards R.S., Sawant A., Schachter C.L. (2011). Exercise Therapy for Fibromyalgia. Curr. Pain Headache Rep..

[5] Bidonde J., Busch A., Bath B., Milosavljevic S. (2014). Exercise for Adults with Fibromyalgia: An Umbrella Systematic Review with Synthesis of Best Evidence. Curr. Rheumatol. Rev..

[6] Macfarlane G.J., Kronisch C., Dean L.E., Atzeni F., Häuser W., Fluß E., Choy E., Kosek E., Amris K., Branco J. (2017). EULAR revised recommendations for the management of fibromyalgia. Ann. Rheum. Dis..

[7] Gusi N., Parraca J., Adsuar, Olivares P., Penacho A., Rivera J., Pastor M.A., Gusi N. (2009). Physical exercise and Fibromyalgia. Physical Exercise Guidelines for People with Fibromyalgia.

[8] Mannerkorpi K., Nordeman L., Cider Å., Jonsson G. (2010). Does moderate-to-high intensity Nordic walking improve functional capacity and pain in fibromyalgia? A prospective randomized controlled trial. Arthritis Res. Ther..

[9] López-Roig S., Pastor M.A., Peñacoba C., Lledó A., Sanz Y., Velasco F. (2016). Prevalence and predictors of unsupervised walking and physical activity in a community population of women with fibromyalgia. Rheumatol. Int..

[10] Room J., Hannink E., Dawes H., Barker K. (2017). What interventions are used to improve exercise adherence in older people and what behavioural techniques are they based on? A systematic review. BMJ Open.

[11] Pastor-Mira M.-A., López-Roig S., Peñacoba C., Sanz-Baños Y., Lledó A., Velasco L. (2020). Predicting walking as exercise in women with fibromyalgia from the perspective of the theory of planned behavior. Women Health.

[12] Peñacoba C., Pastor M.-Á., López-Roig S., Velasco L., Lledo A. (2017). Walking Beliefs in Women With Fibromyalgia: Clinical Profile and Impact on Walking Behavior. Clin. Nurs. Res..

[13] Suso-Ribera C., García-Palacios A., Botella C., Ribera-Canudas M.V. (2017). Pain Catastrophizing and Its Relationship with Health Outcomes: Does Pain Intensity Matter?. Pain Res. Manag..

[14] Wolfe F., Clauw D.J., Fitzcharles M.A., Goldenberg D.L., Katz R.S., Mease P., Russell A.S., Russell I.J., Winfield J.B., Yunus M.B. (2010). The American College of Rheumatology preliminary diagnostic criteria for fibromyalgia and measurement of symptom severity. Arthritis Care Res..

[15] Cleeland C.S., Ryan K.M. (1994). Pain assessment: Global use of the Brief Pain Inventory. Ann. Acad. Med. Singapore.

[16] Dworkin R.H., Turk D.C., Farrar J.T., Haythornthwaite J.A., Jensen M.P., Katz N.P., Kerns R.D., Stucki G., Allen R.R., Bellamy N. (2005). Core outcome measures for chronic pain clinical trials: IMMPACT recommendations. Pain.

[17] Salgueiro M., García-Leiva J.M., Ballesteros J., Hidalgo J., Molina R., Calandre E.P. (2013). Validation of a Spanish version of the Revised Fibromyalgia Impact Questionnaire (FIQR). Health Qual. Life Outcomes.

[18] Herrero M.J., Blanch J., Peri J.M., De Pablo J., Pintor L., Bulbena A. (2003). A validation study of the hospital anxiety and depression scale (HADS) in a Spanish population. Gen. Hosp. Psychiatry.

[19] García Campayo J., Rodero B., Alda M., Sobradiel N., Montero J., Moreno S. (2008). Validation of the Spanish version of the Pain Catastrophizing Scale in fibromyalgia. Med. Clin. (Barc).

[20] Romero-Moreno R., Losada A., Fernández-Fernández V., Márquez-González M., Gillanders D. (2014). Cognitive fusion in dementia caregiving: Psychometric properties of the spanish version of the “cognitive fusion questionnaire”. Behav. Psychol. Psicol. Conduct..

[21] IBM Corp (2017). IBM Corp IBM SPSS Statistics for Windows.

[22] Hayes A.F. (2017). Introduction to Mediation, Moderation, and Conditional Process Analysis Second Edition A Regression-Based Approach.

[23] Ferguson C.J. (2016). An effect size primer: A guide for clinicians and researchers. Methodological Issues and Strategies in Clinical Research.

[24] Ellis A. (2010). Overcoming Destructive Beliefs, Feelings, and Behaviors: New Directions for Rational Emotive Behavior Therapy.

[25] Bendayan R., Esteve R., Blanca M.J. (2012). New empirical evidence of the validity of the Chronic Pain Acceptance Questionnaire: The differential influence of activity engagement and pain willingness on adjustment to chronic pain. Br. J. Health Psychol..

[26] Fish R.A., McGuire B., Hogan M., Morrison T.G., Stewart I. (2010). Validation of the Chronic Pain Acceptance Questionnaire (CPAQ) in an Internet sample and development and preliminary validation of the CPAQ-8. Pain.

[27] Gatchel R., McGeary D., McGeary C., Lippe B. (2014). Interdisciplinary chronic pain management: Past, present, and future. Am. Psychol..

[28] Angarita-Osorio N., Pérez-Aranda A., Feliu-Soler A., Andrés-Rodríguez L., Borràs X., Suso-Ribera C., Slim M., Herrera-Mercadal P., Fernández-Vergel R., Blanco M.E. (2019). Patients With Fibromyalgia Reporting Severe Pain but Low Impact of the Syndrome: Clinical and Pain-Related Cognitive Features. Pain Pract..

[29] Montoro C.I., Reyes del Paso G.A. (2015). Personality and fibromyalgia: Relationships with clinical, emotional, and functional variables. Pers. Individ. Dif..

[30] Peñacoba Puente C., Velasco Furlong L., Écija Gallardo C., Cigarán Méndez M., McKenney K. (2013). Anxiety, Depression and Alexithymia in Fibromyalgia: Are There Any Differences According to Age?. J. Women Aging.

[31] Asmundson G.J., Norton P.J., Vlaeyen J.W. (2004). Fear-avoidance models of chronic pain: An overview. Understanding and Treating Fear of Pain.

[32] McCracken L.M. (2013). Committed action: An application of the psychological flexibility model to activity patterns in chronic pain. J. Pain.

[33] McCracken L.M., Vowles K.E. (2014). Acceptance and commitment therapy and mindfulness for chronic pain: Model, process, and progress. Am. Psychol..

[34] Eisele A., Schagg D., Krämer L.V., Bengel J., Göhner W. (2019). Behaviour change techniques applied in interventions to enhance physical activity adherence in patients with chronic musculoskeletal conditions: A systematic review and meta-analysis. Patient Educ. Couns..

[35] Michie S., Van Stralen M.M., West R. (2011). The behaviour change wheel: A new method for characterising and designing behaviour change interventions. Implement. Sci..

[36] Meade L.B., Bearne L.M., Sweeney L.H., Alageel S.H., Godfrey E.L. (2019). Behaviour change techniques associated with adherence to prescribed exercise in patients with persistent musculoskeletal pain: Systematic review. Br. J. Health Psychol..

[37] Eynon M., Foad J., Downey J., Bowmer Y., Mills H. (2019). Assessing the psychosocial factors associated with adherence to exercise referral schemes: A systematic review. Scand. J. Med. Sci. Sports.

